# Case of Femoral Hernia

**Published:** 1846-09

**Authors:** George Holmes

**Affiliations:** Perth, Canada West


*Case of Femoral Hernia.* By George Holmes, of Perth, Canada West.—In 1840, I was visiting one of the back townships, about 30 miles from home, when I heard that a poor old man, residing a short distance from the house where I was stopping, was suffering from obstinate constipation, and was considered by his neighbours to be in a dying state. I volunteered my services, which were thankfully accepted. On my arrival at his house, I found the patient, who was 63 years of age, in great suffering; the bowels had not been relieved for five days, and he was then vomiting stercoraceous matter, pulse 130, and occasional hiccup. On examination, I found a tumour, about the size of a small hen's egg, in the groin, in the situation generally occupied in femoral hernia; rather tender on pressure, but elastic, and increasing in size on the patient's coughing; the abdomen tense, and rather tender. He gave the following history of the case:—When about 30 years of age, he had a violent attack of whooping-cough, and, after a severe paroxysm, felt a swelling rise suddenly in his groin. This soon disappeared, but returned again at intervals. He consulted a medical man in the United States, where he was residing at the time, who gave him a truss, which he wore for eight years constantly, when he left it off, as the tumour did not return, and to all appearance he was cured. Indeed, it did not again show itself until five days previous to my seeing him, after a lapse of 25 years.

I bled him, used the hot bath, and applied the taxis, but all in vain. The symptoms being so unfavourable, and the case appearing so manifestly urgent, I determined to lose no time, but to operate. To my extreme mortification, I discovered that I had left my pocket-case at home. What was to be done? There was no medical aid within 20 miles, and to send to my own house in the bad state of the roads would occupy too much time; indeed, the delay would have been fatal. I had in my pocket a sharp, long-bladed pen-knife, and with this I determined to hazard an operation—having first made a director out of a piece of hard wood. I commenced by making an incision through the skin over the tumour, and then a transverse one at its base, forming an inverted T. Having dissected back the flaps, and divided the cellular and fascial covering, and exposed the hernial sac, I found, on opening it, a large fold or knuckle of intestine of a dark brown colour, and of a very glazy appearance. The sac contained very little serum. The next step was to insert my director under the stricture which was very high up, and having guarded my penknife, by rolling some thread round it to make it resemble a hernia bistoury, passed it upon its wooden guide, and divided the stricture with very little difficulty, and returned the intestine. Very little blood was lost; the wound was closed and the patient put to bed. About an hour afterwards I administered an enema, which thoroughly emptied the bowels and having left the poor man an opiate, I consigned him to the care of an old midwife, hardly expecting a recovery. About a month afterwards I had the satisfaction to hear that he was quite convalescent; and I afterwards saw him—about two years after the operation—in very good health and quite free from any hernial swelling at all.

I may here remark, that one very singular feature in this case is, that it was a crural or femoral hernia occurring in the male. Of this there can be no doubt, as the tumour began at the edge of Poupart's ligament and then proceeded downwards between the crural vessels and the os pubis, and the abdominal ring, and the parts above the ligament, could be distinctly felt, perfectly uncovered by the hernia. This form is very rare in the male, although Lawrence, in his work on "Hernia," states his belief, that it is not so uncommon as many authors consider it. It is the first instance that has ever come under my observation, either in private or public practice, although during a period of nine years I was constantly in attendance in some of the largest hospitals both in England and Ireland, where operations for hernia were of frequent occurrence.—*British American Journal.*

